# Upregulation of *BCAM* and its sense lncRNA *BAN* are associated with gastric cancer metastasis and poor prognosis

**DOI:** 10.1002/1878-0261.12638

**Published:** 2020-02-13

**Authors:** Juan Jin, Shanshan Xie, Qiang Sun, Zhenxia Huang, Kanghua Chen, Dongyang Guo, Xianping Rao, Yujie Deng, Yiman Liu, Shuang Li, Wenyu Cui, Valentina Chanu Maibam, Junni Wang, Wei Zhuo, Tianhua Zhou

**Affiliations:** ^1^ Department of Cell Biology and Department of Gastroenterology of Sir Run Run Shaw Hospital Zhejiang University School of Medicine Hangzhou China; ^2^ The Children’s Hospital Zhejiang University School of Medicine Hangzhou China; ^3^ The First People’s Hospital of Xiaoshan District Hangzhou China; ^4^ Kidney Disease Center of the First Affiliated Hospital Zhejiang University School of Medicine Hangzhou China; ^5^ Institute of Gastroenterology Zhejiang University Hangzhou China; ^6^ Collaborative Innovation Center for Diagnosis and Treatment of Infectious Diseases Hangzhou China; ^7^ Department of Molecular Genetics University of Toronto ON Canada

**Keywords:** BCAM, gastric cancer, lncRNA, metastasis, prognosis

## Abstract

Patients with metastatic gastric cancer (GC) have a poor prognosis; however, the molecular mechanism of GC metastasis remains unclear. Here, we employed bioinformatics to systematically screen the metastasis‐associated genes and found that the levels of basal cell adhesion molecule (*BCAM*) were significantly increased in GC tissues from patients with metastasis, as compared to those without metastasis. The upregulation of *BCAM* was also significantly associated with a shorter survival time. Depletion of BCAM inhibited GC cell migration and invasion. Knockout (KO) of *BCAM* by the CRISPR/Cas9 system reduced the invasion and metastasis of GC cells. To explore the mechanism of *BCAM* upregulation, we identified a previously uncharacterized *BCAM* sense lncRNA that spanned from exon 6 to intron 6 of *BCAM*, and named it as *BCAM*‐associated long noncoding RNA (*BAN*). Knockdown of *BAN* inhibited BCAM expression at both mRNA and protein levels. Knockdown of *BAN* suppressed GC cell migration and invasion, which was effectively rescued by ectopic expression of *BCAM*. Further clinical data showed that *BAN* upregulation was associated with GC metastasis and poor prognosis. Importantly, *BAN* expression was also significantly associated with that of *BCAM* in GC tissues. Taken together, these results indicate that increased expression of *BCAM* and its sense lncRNA *BAN* promote GC cell invasion and metastasis, and are associated with poor prognosis of GC patients.

## Graphical abstract

We found that the levels of *BCAM* and its sense lncRNA *BAN* were increased in gastric cancer (GC) tissues with metastasis. Knockdown of *BAN* inhibited BCAM expression at both mRNA and protein levels. Moreover, increased expression of *BCAM* and its sense lncRNA *BAN* promote GC cell invasion and metastasis, and are associated with poor prognosis of GC patients.

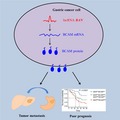

**Abbreviations**
ACRG: Asian cancer research group
AJCC: American Joint Committee on Cancer
*BAN*: BCAM‐associated lncRNA
BCAM: basal cell adhesion molecule
FACS: fluorescence‐activated cell sorting
GAPDH: glyceraldehyde‐3‐phosphate dehydrogenase
GC: gastric cancer
lncRNA: long noncoding RNA
MTT: 3‐(4,5‐Dimethylthiazol‐2‐yl)‐2,5‐diphenyltetrazolium bromide
qRT‐PCR: quantitative reverse transcription PCR
ROC: receiver operator characteristic
TCGA: the cancer genome atlas

## 1 Introduction

Gastric cancer (GC) is the fifth most frequently diagnosed cancer and is the third leading cause of cancer‐related deaths worldwide (Bray *et al.*, 2018). Due to the lack of obvious symptoms and biomarkers for early‐stage GC, ~ 40% of GC patients present with metastasis at the time of diagnosis (Bernards *et al.*, 2013). Moreover, the overall survival of GC patients with metastasis is poor, with an about 5% of the 5‐year survival rate (Bernards *et al.*, 2013). Thus, it is in an emergency to clarify the molecular mechanisms of GC metastasis.

Basal cell adhesion molecule (BCAM), also known as Lutheran, is widely expressed in various tissues and is involved in many biological processes, such as cell adhesion, migration, and invasion (Bartolini *et al.*, 2016; Campbell *et al.*, 1994; De Grandis *et al.*, 2013; El Nemer *et al.*, 1998; Gauthier *et al.*, 2005; Hines *et al.*, 2003; Kikkawa and Miner, 2005; Kikkawa *et al.*, 2013; Parsons *et al.*, 1995). Emerging studies have shown that BCAM plays an important role in tumor progression, including skin tumors, hepatocellular carcinoma, colorectal cancer, and bladder cancer (Bartolini *et al.*, 2016; Campbell *et al.*, 1994; Chang *et al.*, 2017; Drewniok *et al.*, 2004; Kikkawa *et al.*, 2013). However, the role of BCAM in GC progression is still unclear.

Long noncoding RNA, more than 200 nt in length, are transcripts without protein‐coding capacity (Derrien *et al.*, 2012; Djebali *et al.*, 2012; Ma *et al.*, 2013). Increasing data demonstrate that lncRNA regulate gene expression through diverse mechanisms, including gene activation and suppression, chromatin modification and remodeling, splicing and translation modulation, acting as miRNA sponges, and small RNA precursors (Ponting *et al.*, 2009; Spitale *et al.*, 2011; Wang and Chang, 2011; Wilusz *et al.*, 2009). Accumulating evidence has shown that lncRNA play key roles in the formation and progression of many cancers, including GC (Gutschner and Diederichs, 2012; Spizzo *et al.*, 2012; Xie *et al.*, 2016). lncRNA are important regulators of cell proliferation, apoptosis, migration, and differentiation, and dysregulated lncRNA result in tumor growth, invasion, and metastasis (Gupta *et al.*, 2010). Emerging data demonstrate that lncRNA, including *ANRIL*, *FENDRR*, *GAS5, GHET1*, *GMAN*, *MALAT1*, and *PVT1*, are involved in GC progression (Kong *et al.*, 2015; Sun *et al.*, 2014; Tripathi *et al.*, 2010; Xie *et al.*, 2016; Xu *et al.*, 2014; Yang *et al.*, 2014; Zhang *et al.*, 2014; Zhuo *et al.*, 2019). Our recent study shows that *GMAN,* upregulated in GC tissues, is associated with metastasis and promotes the expression of ephrin A1 (Zhuo *et al.*, 2019).

In this study, we found that *BCAM* expression was significantly correlated with GC metastasis and poor prognosis. KO of *BCAM* suppressed GC cell invasion and metastasis. Furthermore, we identified a previously undescribed gene BCAM‐associated lncRNA (*BAN*) as a sense lncRNA of *BCAM*, which was also associated with GC metastasis and poor prognosis. Knockdown of *BAN* not only inhibited BCAM expression, but also suppressed GC cell invasion, which was successfully rescued by ectopic expression of BCAM. Thus, our data suggest that *BCAM* and its sense lncRNA *BAN* play a crucial role in GC metastasis.

## 2 Materials and methods

### 2.1 Bioinformatics analysis

RNA‐seq data of The Cancer Genome Atlas (TCGA) cohort were downloaded from the Genomic Data Commons data portal (url) (Cancer Genome Atlas Research, 2014). R DESeq2 package was used to find genes with differential expression level between GC tissues with distant metastasis and those without metastasis, and the genes with FDR under 0.05 and expression fold change over 1.8 were considered as significantly upregulated genes (Love *et al.*, 2014). Microarrays of Asian Cancer Research Group (ACRG) cohort were obtained from Gene Expression Omnibus database (url) (Cristescu *et al.*, 2015). The raw CEL files were normalized with the RMA algorithm using Custom chip Definition Files mapping to official Gene Symbol (Brainarray v.22 http://brainarray.mbni.med.umich.edu/) (Manhong *et al.*, 2005). Notably, the averaged expression was calculated for genes with multiple targeted probes. R limma package was used to find the differentially expressed genes between GC tissues with distant metastasis and those without metastasis, and the genes with FDR under 0.05 and expression fold change over 1.8 were considered as significantly upregulated genes (Ritchie *et al.*, 2015). Univariate Cox regression analysis was applied to identify the survival‐related genes in TCGA and ACRG cohorts, and genes with *P* value under 0.05 and *Z*‐score over 0 were considered as adversely prognostic.

### 2.2 Human tissue samples

All GC tissue samples were obtained from GC patients undergoing gastrectomy with informed consent. Zhejiang cohort (*n* = 64) samples were collected from Sir Run Run Shaw Hospital, Zhejiang University School of Medicine (Hangzhou, China) and Zhejiang Cancer Hospital (Hangzhou, China). Among them, 11 pairs of GC tissues were from patients with distant metastasis and age‐ and sex‐matched patients without metastasis. Ethical consent was granted from the Ethical Committee Review Board of Zhejiang University School of Medicine. The study methodologies conformed to the standards set by the Declaration of Helsinki.

### 2.3 Cell culture

Human GC cell line BGC‐823 was obtained from the Chinese Academy of Sciences (Jiao *et al.*, 2014). Human GC cell line SGC‐7901 was obtained from Beijing Cancer Hospital (Xing *et al.*, 2012). BGC‐823 and SGC‐7901 cells were maintained in RPMI‐1640 medium supplemented with 10% FBS (Gibco BRL, Grand Island, NY, USA) with 5% CO_2_. All cell lines were routinely tested negative for mycoplasma.

### 2.4 RNA extraction and quantitative RT‐PCR

Total RNA was extracted from human tissue samples and cultured cells, respectively, using the TRIzol™ Reagent (Invitrogen, Carlsbad, CA, USA) following the manufacture’s protocol. The concentration and quality of RNA were determined with a NanoDrop spectrophotometer (NanoDrop Technologies, Thermo Fisher Scientific, Waltham, MA, USA) and gel analysis. Reverse transcription reactions were carried out using High‐Capacity cDNA Reverse Transcription Kits (Applied Biosystems, Foster City, CA, USA). LightCycler® 480 Probes Master (Roche, Basel, Switzerland) was used to evaluate the expression of *BCAM* and *BAN* in human tissue samples and cultured cells. The relative expression of *BCAM* and *BAN* was calculated using glyceraldehyde‐3‐phosphate dehydrogenase (GAPDH) as the endogenous control to normalize the data. The sequences of the primers used are as follows: *BCAM*, sense 5′‐GTGCTTTCCTTACCTCTAA‐3′ antisense 5′‐GTAGGTGCCATTGGAATC‐3′ and probe 5′‐AGTCGTGAACTGCTCCGTGC‐3′; *GAPDH*, sense 5′‐GGACCTGACCTGCCGTCTAG‐3′, antisense 5′‐TAGCCCAGGATGCCCTTAG‐3′, and probe 5′‐CCTCCGACGCCTGCTTCACC‐ACCT‐3′; *BAN*, sense 5′‐GACTCTTGACCTATACTCTTAG‐3′, antisense 5′‐TACGGGTCATAGGTTTCA‐3′, and probe 5′‐CAACCTCTGAACTCTGGCACTC‐3′.

### 2.5 Vector construction, siRNA, and transfection

Full‐length human *BCAM* and *BAN* were amplified from the cDNA of BGC‐823 cells and were cloned into pCS2 (+) and pcDNA3.1 vectors, respectively. Both plasmids were confirmed by DNA sequencing. SGC‐7901 cells were then transfected with an empty vector or the BCAM‐expressing plasmid using Lipofectamine™ 2000 (Invitrogen). BGC‐823 cells were transfected with siRNA for BCAM or *BAN* using lipofectamine™ RNAiMAX (Invitrogen). siRNA corresponding to the following sequences for BCAM or *BAN* silencing were synthesized by GenePharma: 5′‐CAACGUGUUUGCAAAGCCATT‐3′ for siBCAM‐1, 5′‐CUGUCGCUCAGUUCUAUCATT‐3′ for siBCAM‐2, 5′‐CUCUGGCACUCAGAAUAAUTT‐3′ for si*BAN*‐1, and 5′‐GUUUAUGACUAAAUGGUGCTT‐3′ for si*BAN*‐2.

### 2.6 SDS/PAGE and immunoblot

Cells were lysed using RIPA protein extraction reagent (Beyotime, Shanghai, China) supplemented with a protease inhibitor cocktail (Roche). The cell lysates were separated by SDS/PAGE and were transferred onto a poly(vinylidene difluoride) membrane. The membranes were incubated with 5% BSA. The proteins were detected using an anti‐BCAM monoclonal antibody (1 : 1000; Abcam, Cambridge, MA, USA), anti‐GAPDH (1 : 1000; Sigma‐Aldrich, San Francisco, CA, USA), and anti‐ACTIN (1 : 1000; Sigma‐Aldrich).

### 2.7 MTT assay

The 3‐(4, 5‐dimethylthiazol‐2‐yl)‐2, 5‐diphenyl‐tetrazoliumbromide (MTT) assay was performed at 0, 24, 48, and 72 h post‐transfection. The cells in the 96‐well culture were incubated with MTT (5 mg·mL^−1^, 20 μL) for 4 h. After that, 150 μL of DMSO was added and resuspended until the cysts were completely dissolved. The absorbances of samples were measured with a spectrophotometer at 490 nm. Each assay was performed in triplicate and was independently repeated three times.

### 2.8 Colony formation assay

For the colony formation assay, transfected cells (*n* = 500) were placed in 6‐well plates. After 2 weeks, the cells were fixed with 4% paraformaldehyde and were stained with 0.5% crystal violet in 20% EtOH for 15 min. Visible colonies were photographed and counted by imagej software (NIH, Bethesda, MD, USA). Each assay was performed in triplicate and was independently repeated three times.

### 2.9 FACS assay

A fluorescence‐activated cell sorting (FACS) assay was performed to analyze the cell cycle distribution. The cells were collected and fixed with 70% EtOH overnight. Then, the fixed cells were treated with propidium iodide and subjected to cell cycle distribution analysis using a flow cytometer (Beckman, Brea, CA, USA).

### 2.10 Transwell assay

For the transwell assay, 5 × 10^4^ cells were placed in the top chamber in medium with 1% FBS, and the medium supplemented with 20% FBS was filled in the lower chamber and served as a chemoattractant. After incubation, the cells on the lower surface of the membrane were stained with crystal violet and were counted by imagej software. Each assay was performed in triplicate and was independently repeated three times.

### 2.11 Matrigel invasion assay

For the invasion assay, 5 × 10^4^ cells were placed in the top chamber with a Matrigel‐coated membrane (24‐well insert; pore size, 8 mm; BD Biosciences, New York, NY, USA) in medium with 1% FBS, and medium supplemented with 20% FBS was filled in the lower chamber and used as a chemoattractant. After incubation, the cells on the lower surface of the membrane were stained with crystal violet and were counted by imagej software. Each assay was performed in triplicate and was independently repeated three times.

### 2.12 Knockout of *BCAM*

The pX330 vector (Addgene plasmid 42230) was a gift from F. Zhang. Plasmids expressing hCas9 and sgRNA for *BCAM* were prepared by ligating oligos into the BbsI site of pX330. The sequences used for sgRNA are as follows: sense: 5′‐ ACCGCATGGAGCCCCCGGACGCAC‐3′, and antisense: 5′‐ AACGTGCGTCCGGGGGCTCCATGC‐3′. This plasmid was designated pX330‐BCAM. Then, the plasmid was introduced into BGC‐823 cells and treated with puromycin at 48 h after transfection. After 48 h, the cells were placed into 96‐well plates at the concentration of 1 cell/well. Single colonies were picked and validated by genotyping and immunoblot analysis.

### 2.13 Tumor metastasis model

Nude mice (6–8 weeks old) were maintained under SPF conditions with individually ventilated cages in the Animal Facility of Zhejiang University. The spleens of the mice were inoculated with 10^6^ BGC‐823 cells. Three weeks later, the livers were harvested, and external areas of metastatic masses were quantified. Animal experiments were approved by the Institutional Animal Care and Use Committee of Zhejiang University.

### 2.14 Statistical analysis

The significance of the differences between groups was estimated by the Student’s *t*‐test or χ^2^ test as appropriate. *P* < 0.05 was considered to be statistically significant. Kaplan–Meier method with the log‐rank test was adopted to evaluate the overall survival of different groups. Univariate and multivariate Cox proportional hazards models were performed using R survival package. Receiver operating characteristic and prediction error (PE) curves were produced using the survcomp and PEC package, respectively. All the above analysis was conducted using r software (version 3.4.4, http://cran.r-project.org).

## 3 Results

### 3.1 *BCAM* upregulation is associated with GC metastasis and poor prognosis

**Figure 1:**
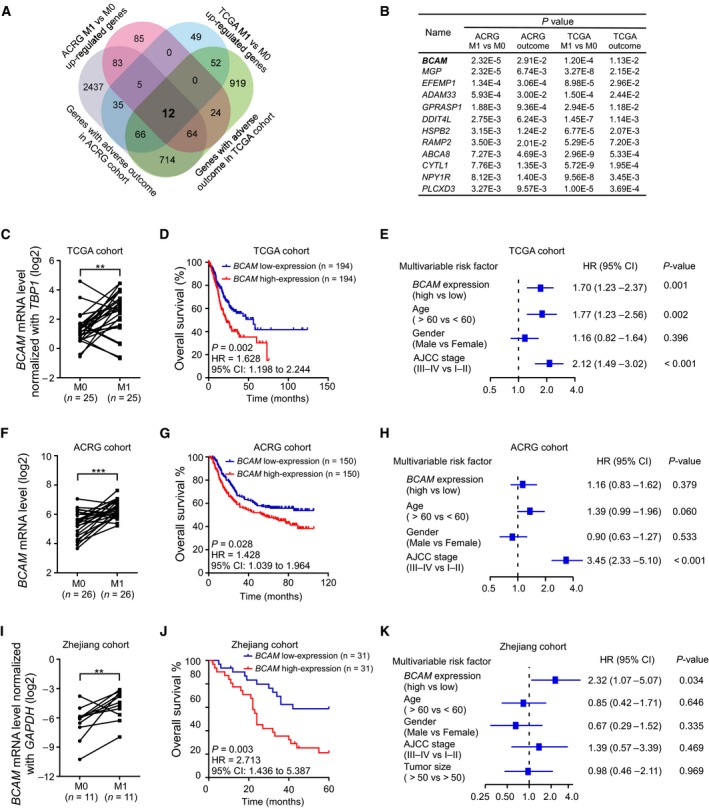
*BCAM* upregulation is associated with GC metastasis and poor prognosis. (A) The Venn diagram illustrating the number of upregulated genes in GC tissues with distant metastasis that had a reverse outcome in both TCGA and ACRG cohorts. (B) Twelve genes that were upregulated in metastatic GC tissues and were positively associated with a poor prognosis for GC patients were listed. (C) The relative expression of *BCAM* in 25 GC tissues with distant metastasis (M1) compared to that in age‐ and sex‐matched GC tissues without metastasis (M0) from the TCGA dataset. The results are presented as log_2_ FPKM values normalized to that of *TBP1*. **P < 0.01. (D) The relative expression of *BCAM* in 26 GC tissues with distant metastasis (M1) compared to that in age‐ and sex‐matched GC tissues without metastasis (M0) from the ACRG cohort. ***P < 0.001. (E) Kaplan–Meier survival curve analysis between patients with *BCAM*‐high expression group (*n* = 194) and *BCAM*‐low expression group (*n* = 194) in TCGA cohort. The GC patients were classified into *BCAM*‐high or *BCAM*‐low expression groups according to the median value. *P* = 0.002, HR = 1.628, 95% CI: 1.198–2.244. (F) Kaplan–Meier survival curve analysis between patients with *BCAM*‐high expression group (*n* = 150) and *BCAM*‐low expression group (*n* = 150) in the ACRG cohort. The GC patients were classified into *BCAM*‐high or *BCAM*‐low expression groups according to the median value. *P* = 0.028, HR = 1.428, 95% CI: 1.039–1.964. (G) The forest plot depicted the multivariable Cox analysis results of *BCAM* in the TCGA cohort. All the bars correspond to 95% confidence intervals. (H) The forest plot depicted the multivariable Cox analysis results of *BCAM* in the ACRG cohort. All the bars correspond to 95% confidence intervals. (I) Quantitative RT‐PCR was performed to analyze the relative expression of *BCAM* expression in 11 GC tissues with distant metastasis (M1) compared to that in age‐ and sex‐matched GC tissues without metastasis (M0). The results are presented as fold changes based on log_2_ values normalized to *GAPDH*. **P < 0.01. (J) Kaplan–Meier survival curve analysis between patients with *BCAM*‐high expression group (*n* = 31) and *BCAM*‐low expression group (*n* = 31) in the Zhejiang cohort. The GC patients were classified into *BCAM*‐high or *BCAM*‐low expression groups according to the median value. *P* = 0.003, HR = 2.713, 95% CI: 1.436–5.387. All the bars correspond to 95% confidence intervals. (K) The forest plot depicted the multivariable Cox analysis results of *BCAM* in the Zhejiang cohort. All the bars correspond to 95% confidence intervals.

To systematically screen GC metastasis‐associated genes, we first performed differential expression analysis between GC tissues with distant metastasis and those without metastasis in two cohorts from TCGA and ACRG datasets. Seventeen genes were found to be upregulated in GC tissues with metastasis (*P* < 0.05). These genes were further filtered by analysis of association with the shorter survival times of GC patients (*P* < 0.05). A Venn diagram revealed that 12 overlapping genes were upregulated in GC tissues with metastasis and associated with poor prognosis (Fig. 1A). *BCAM* was among the genes with lowest *P* value (Fig. 1B). Further paired statistical analysis confirmed that *BCAM* was significantly upregulated in GC tissues with metastasis compared to those without metastasis (Fig. 1C,D). *BCAM* upregulation was associated with poor prognosis of GC patients in both TCGA and ACRG cohorts (Fig. 1E,F). Multivariate Cox analysis showed that high expression of *BCAM* was independently associated with reduced overall survival time from TCGA cohort, but not ACRG cohort (Fig. 1G,H).

To verify these bioinformatics results, we collected 62 GC tissues from Sir Run Run Shaw Hospital, Zhejiang University School of Medicine and Zhejiang Cancer Hospital with informed consent (Zhejiang cohort). Quantitative real‐time RT‐PCR (qRT‐PCR) analysis revealed that the levels of *BCAM* were significantly increased in GC tissues with metastasis compared to those in tissues without metastasis (Fig. 1I). Importantly, Kaplan–Meier curve analysis of this cohort (*n* = 62) showed that the upregulation of *BCAM* was significantly associated with the poor overall survival of GC patients (Fig. 1J). Further multivariate Cox analysis confirmed that *BCAM* expression was an independent predictor for predicting clinical outcome of GC patients (Fig. 1K).

### 3.2 Knockdown of BCAM suppresses GC cell migration and invasion

**Figure 2:**
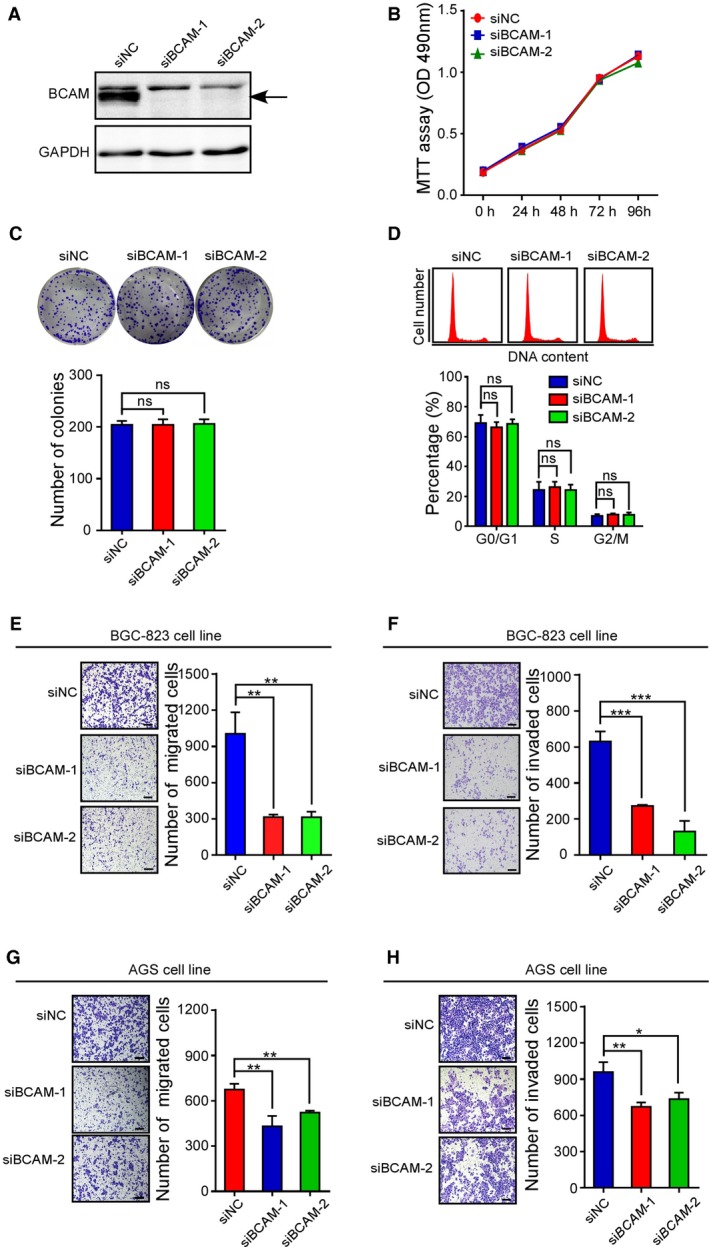
Knockdown of BCAM expression suppresses GC cell migration and invasion. (A) Immunoblot analysis of the BCAM expression levels following the treatment of BGC‐823 cells with scrambled siRNA and siBCAM. (B–D) MTT assay (B), colony‐forming growth assay (C), and cell cycle (D) analysis were performed using BGC‐823 cells with the indicated treatment. Colonies were captured and counted. The bar chart represents the percentage of cells in G0/G1, S, or G2/M phase, as indicated. (E, F) Transwell migration (E) and Matrigel invasion assays (F) were carried out using GC cells with the indicated treatments. (G, H) Transwell migration (G) and Matrigel invasion assays (H) were carried out using AGS cells with the indicated treatments. Migrated and invaded cells were counted. Scar bar, 100 μm. Experiments were performed in triplicate. Data are presented as the means ± SDs; ns, no significance; *P < 0.05, **P < 0.01, ***P < 0.001.

To investigate the potential role of BCAM in GC cells, we first performed qRT‐PCR analysis and found that *BCAM* was upregulated at high levels in AGS, MKN‐45, and BGC‐823 cells, and low levels in SGC‐7901, MKN‐74, MGC80‐3, and HGC‐27 cells (Fig. S1A). Then, we depleted BCAM expression by introducing specific siRNA into BGC‐823 cells. The knockdown efficiency was confirmed by a western blot analysis (Fig. 2A). Neither the MTT assay nor the clone formation assay showed that knockdown of BCAM had a significant effect on GC cell proliferation (Fig. 2B,C). FACS analysis showed that silencing BCAM had no significant effect on GC cell cycle progression (Fig. 2D). However, the transwell migration assay and Matrigel invasion assay revealed that knockdown of BCAM dramatically suppressed GC cell migration and invasion (Fig. 2E,F). Additionally, the migration assay and invasion assay using AGS GC cell line showed the same results (Fig. 2G,H).

### 3.3 Ectopic expression of BCAM promotes GC cell migration and invasion

**Figure 3:**
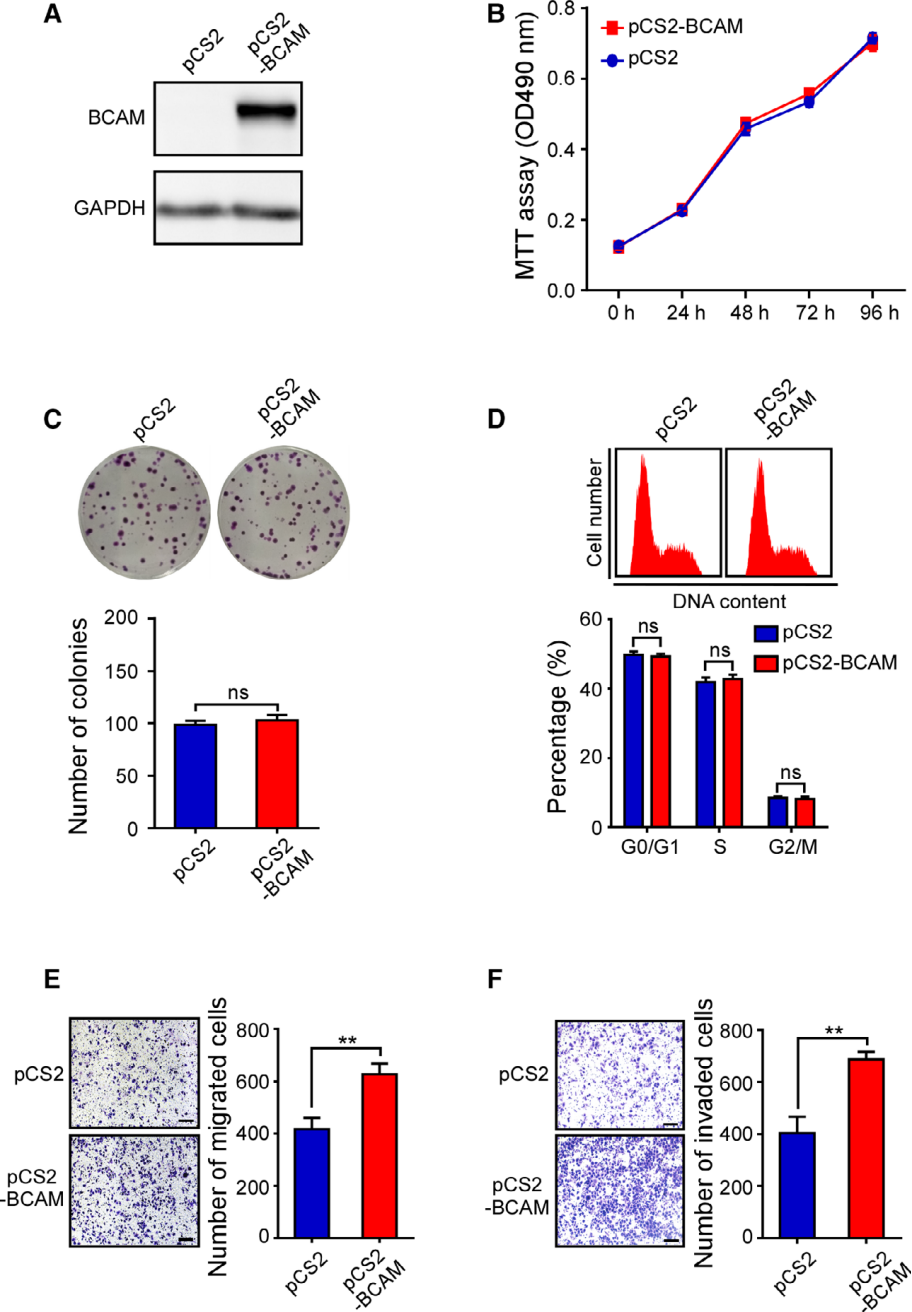
Ectopic expression of BCAM promotes GC cell migration and invasion. (A) Immunoblot analysis of BCAM expression levels following the treatment of SGC‐7901 cells with an empty vector and pCS2‐BCAM. (B–D) SGC‐7901 cells transfected with the indicated plasmids were processed for MTT assay (B), colony‐forming assay (C), and cell cycle analysis (D). The bar chart for cell cycle represents the percentage of cells in the G0/G1, S, or G2/M phase, as indicated. (E, F) Transwell migration (E) and Matrigel invasion assays (F) were carried out using GC cells with the indicated treatments. Migrated and invaded cells were counted. Scar bar, 100 μm. Experiments were performed in triplicate. Data are presented as the means ± SDs; ns, no significance; **P < 0.01.

Next, we overexpressed BCAM expression by introducing pCS2‐BCAM into SGC‐7901 cells. The overexpression efficiency was confirmed by a western blot analysis (Fig. 3A). Consistent with the knockdown of BCAM, the results showed that ectopic expression of BCAM had no significant effect on SGC‐7901 cell proliferation or cell cycle progression (Fig. 3B–D). However, the migration assay and invasion assay showed that ectopic expression of BCAM significantly enhanced GC cell migration and invasion (Fig. 3E,F). Taken together, these data indicate that BCAM is involved in promoting GC cell migration and invasion.

### 3.4 Knockout of *BCAM* by the CRISPR/Cas9 system reduces GC cell metastasis in a mouse model

**Figure 4:**
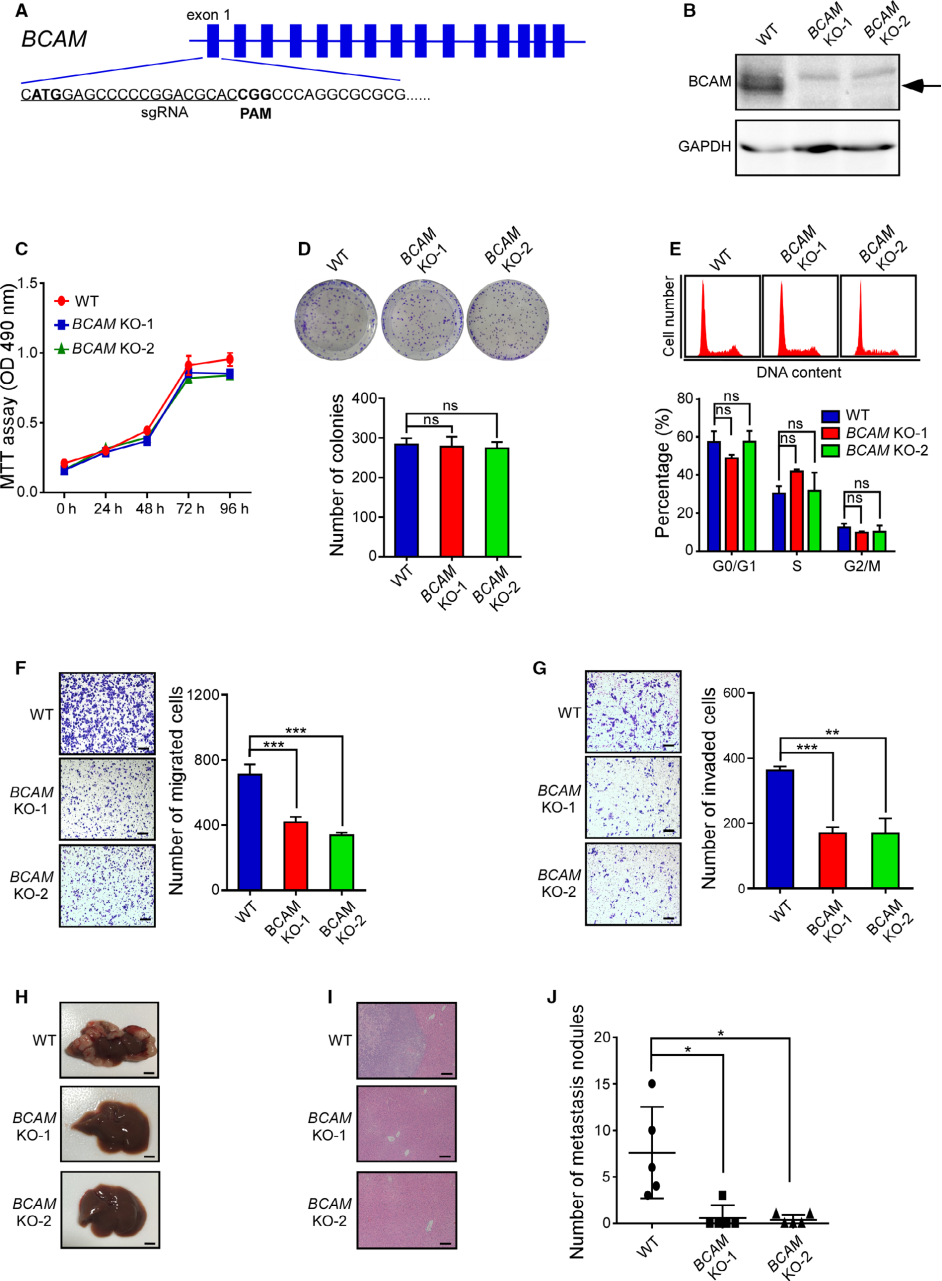
KO of *BCAM* by CRISPR/Cas9 system reduces GC cell metastasis in a mouse model. (A) The *BCAM* KO BGC‐823 cells were produced by the CRISPR/Cas9 system. The schematic diagram of the mutation in *BCAM* locus by CRISPR/Cas9 technique was shown. (B) Immunoblot analysis of the BCAM levels in wild‐type or *BCAM* KO BGC‐823 cells. (C‐G) MTT assay (C), colony‐forming growth assay (D), cell cycle analysis (E), transwell migration (F), and Matrigel invasion analysis (G) of wild‐type or *BCAM* KO BGC‐823 cells. The bar chart for cell cycle represents the percentage of cells in G0/G1, S, or G2/M phase. (H–J) Mice were intrasplenically injected with wild‐type or *BCAM* KO BGC‐823 cells and were subjected to liver metastasis analysis. Representative gross liver (H) and H&E‐stained liver sections (I) from mice were shown. Scar bars, 5 mm (H); Scar bars, 100 μm (I). The liver metastatic nodules were counted (J). Data are presented as the means ± SDs; ns, no significance. *P < 0.05. **P < 0.01. ***P < 0.001.

To further explore the role of BCAM in GC, we used the CRISPR/Cas9 system to knock out the *BCAM* gene in BGC‐823 cells and generated two *BCAM* KO subclones (Fig. 4A). The KO efficiency was confirmed by western blotting (Fig. 4B). Similar to the knockdown of BCAM, *BCAM* KO had no significant effect on cell proliferation, colony formation, or cell cycle distribution (Fig. 4C–E). However, the transwell migration assay and Matrigel invasion assay showed that KO of *BCAM* significantly inhibited GC cell migration and invasion (Fig. 4F,G).

To investigate the potential role of BCAM in GC metastasis in a mouse model, *BCAM* KO cells or wild‐type cells were intrasplenically injected into nude mice, and liver metastases were measured after 6 weeks. The mice inoculated with wild‐type cells developed severe liver metastases, while the injection of *BCAM* KO cells robustly reduced the number of liver metastatic nodules (Fig. 4H–J). Taken together, these results suggest that BCAM plays a critical role in GC metastasis.

### 3.5 BCAM expression is modulated by its associated lncRNA *BAN*

**Figure 5:**
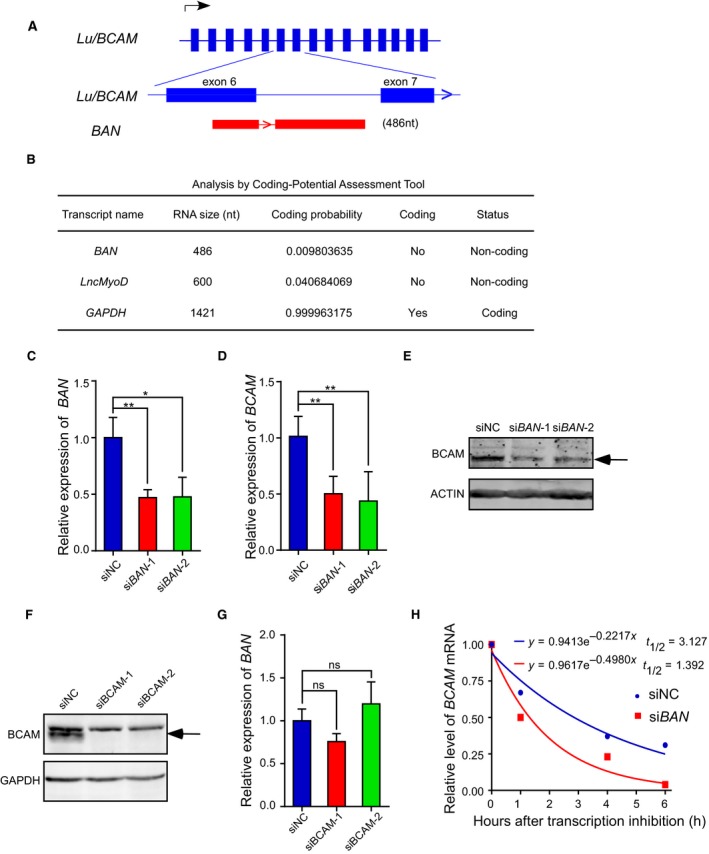
BCAM expression is modulated by its associated sense lncRNA *BAN*. (A) A schematic diagram of the *BCAM* gene locus showing the position of the lncRNA *BAN* within the sixth exon and sixth intron of *BCAM*. Arrows indicate the direction of transcription. (B) The coding potential assessment tool predicted the coding potential of *BAN*. The lncRNA *lncMyoD* and the protein‐coding gene *GAPDH* were also shown. (C) Quantitative RT‐PCR analysis of *BAN* expression levels in BGC‐823 cells transfected with scrambled siRNA and si*BAN*. (D, E) BGC‐823 cells transfected with the indicated siRNA were subjected to qRT‐PCR analysis (D) or immunoblot analysis (E) of BCAM expression. (F) Immunoblot analysis of BCAM expression levels following the treatment of BGC‐823 cells with scrambled siRNA and siBCAM. (G) Quantitative RT‐PCR analysis of *BAN* expression levels in BGC‐823 cells transfected with the indicated siRNA. (H) The effects of *BAN* knockdown on the half‐life (*t*_1/2_) of BCAM mRNA. Data are presented as the means ± SDs; ns, no significance. *P < 0.05. **P < 0.01.

The important role of BCAM in GC prompted us to explore the upstream mechanisms in modulating BCAM expression. We analyzed the *BCAM* gene locus and found a previously uncharacterized *BCAM* sense lncRNA, which we called *BCAM*‐associated long noncoding RNA (*BAN*) (GenBank access ID: AY927517). *BAN*, which spanned from exon 6 and intron 6 of the *BCAM* gene, is a transcript with a length of 486 nt (Fig. 5A). The coding potential calculator and coding potential assessment tool algorithms predicted that *BAN* was a noncoding RNA (Fig. 5B). *BAN* was upregulated at high levels in AGS, MKN‐45, BGC‐823, MKN‐74, and MGC80‐3 cells, and low levels in SGC‐7901 and HGC‐27 cells (Fig. S1B). Consistent with RNA‐FISH assay, nuclear and cytosolic fraction analysis showed that *BAN* was localized both in cytoplasm and nucleus (Fig. S2A,B). lncRNA have been documented to play versatile roles in modulating gene regulation (Sun *et al.*, 2017; Ulitsky and Bartel, 2013). Indeed, knockdown of *BAN* evidently suppressed BCAM expression at both the mRNA and protein levels (Fig. 5C–E), while the depletion of BCAM had no significant effect on *BAN* expression (Fig. 5F,G). Moreover, we performed mRNA stability assay and protein degradation assay to investigate how *BAN* could promote BCAM expression. The results showed that knockdown of *BAN* significantly decreased the half‐life of BCAM mRNA transcripts (Fig. 5H) and BCAM protein (Fig. S3).

### 3.6 BCAM is involved in *BAN*‐mediated invasive activity of GC cells

**Figure 6:**
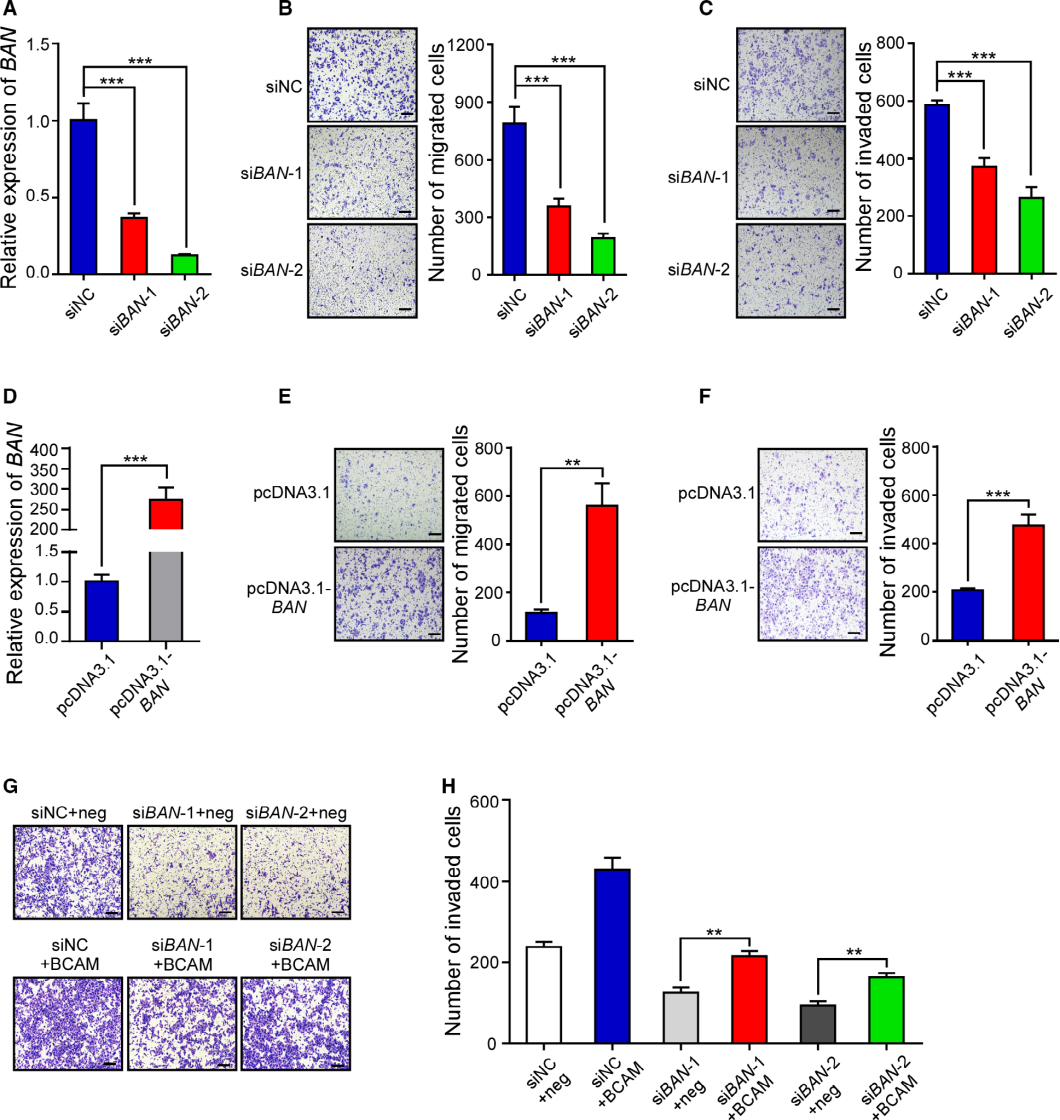
BCAM is involved in *BAN*‐mediated invasive activity of GC cells. (A–C) BGC‐823 cells transfected with the indicated siRNA were subjected to qRT‐PCR analysis (A), transwell migration (B), and Matrigel invasion assays (C). (D–F) SGC‐7901 cells transfected with the indicated plasmids were subjected to qRT‐PCR analysis (D), transwell migration (E), and Matrigel invasion assay (F). (G, H) After knockdown of *BAN*, BGC‐823 cells were transfected with pCS2‐BCAM. Ectopic expression of BCAM rescued the decrease in GC cell invasion induced by the knockdown of *BAN*. Scar bar, 100 μm. Experiments were performed in triplicate. Data are presented as the means ± SDs. **P < 0.01. ***P < 0.001.

To explore the potential role of *BAN* in GC cell migration and invasion *in vitro*, we carried out loss‐ and gain‐of‐function experiments, respectively, by introducing either siRNA specific for *BAN* or pcDNA3.1‐*BAN* into GC cells. The transwell migration assay and Matrigel invasion assay showed that knockdown of *BAN* significantly inhibited GC cell migration and invasion (Fig. 6A–C). Ectopic expression of *BAN* in SGC‐7901 cells dramatically enhanced cell migration and invasion (Fig. 6D–F). It is reasonable to propose that BCAM may be involved in *BAN*‐mediated invasive activity of GC cells after knockdown of *BAN*, BGC‐823 cells were transfected with pCS2‐BCAM. Ectopic expression of BCAM rescued the decreased cell invasion ability caused by knockdown of *BAN* (Fig. 6G,H). The invasion assay suggests that the cotransfection could partially rescue *BAN* RNAi‐decreased GC cell invasion in BGC‐823 cells. These data indicate that *BAN* regulated GC cell migration and invasion by modulating BCAM expression.

### 3.7 *BAN* upregulation is associated with GC metastasis and poor prognosis

**Figure 7:**
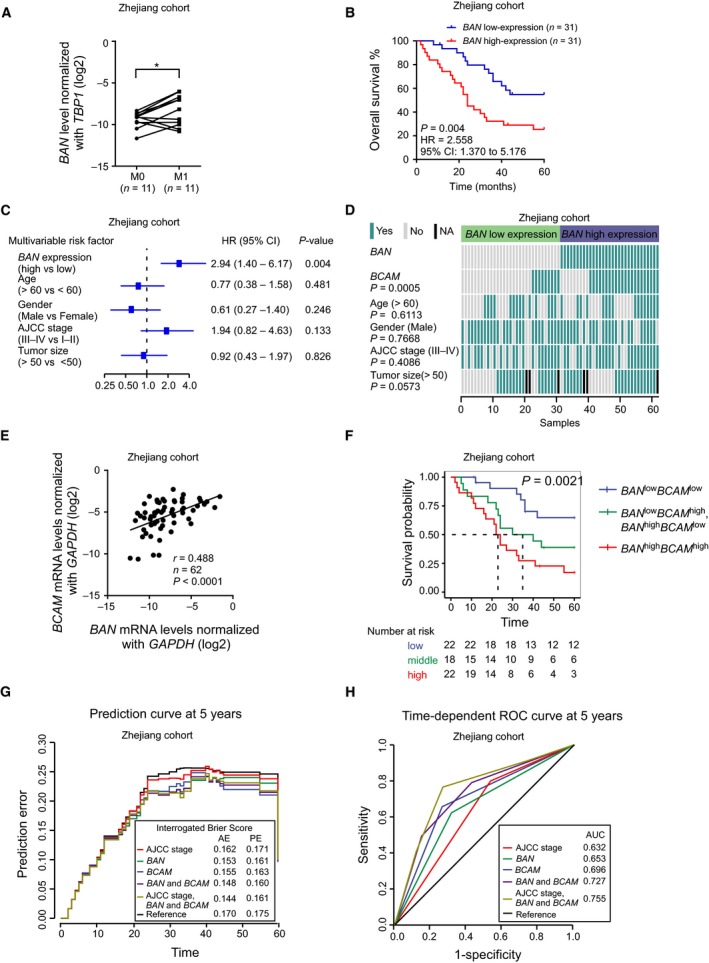
*BAN* upregulation is associated with GC metastasis and poor prognosis. (A) The relative expression of *BAN* expression in 11 GC tissues with distant metastasis (M1) compared to age‐ and sex‐matched GC tissues without metastasis (M0) in the Zhejiang cohort. (B) Kaplan–Meier survival curve analysis between patients with *BAN*‐high expression group and *BAN*‐low expression in the Zhejiang cohort. The GC patients were classified into *BAN*‐high or *BAN*‐low expression groups according to the median value. *P* = 0.004, HR = 2.558, 95% CI: 1.370–5.176. (C) The forest plot depicted the multivariable Cox analysis results of *BAN* in the Zhejiang cohort. All the bars correspond to 95% confidence intervals. (D) The heatmap illustrated the association of *BAN* expression, *BCAM* expression, and different clinical characteristics in the Zhejiang cohort. The GC patients were classified into *BAN*‐high and *BAN*‐low expression groups according to the median value. Statistical significance was performed by the χ^2^ test. (E) The correlation of *BCAM* mRNA and *BAN* expression levels in GC tissues was analyzed by qRT‐PCR. *r* = 0.488, *n* = 62, *P* < 0.0001. (F) Kaplan–Meier survival curve analysis between low (*BCAM‐*low and *BAN*‐low expression group, *n* = 22), middle (*BCAM*‐high and *BAN*‐low expression, or *BCAM*‐low and *BAN*‐high expression group, *n* = 18) and high (*BCAM*‐high and *BAN*‐high expression group, *n* = 22) groups in the Zhejiang cohort. The GC patients were classified into low, middle, and high groups according to the median value of *BCAM* and *BAN*. Number at risk table for *BCAM* and *BAN* expression can also be seen below the plot; *P* value was calculated using the log‐rank test. (G) PE curves of the different predictors in the Zhejiang cohort. Apparent error and 10‐fold cross‐validated cumulative PE at 5 years were computed using Kaplan–Meier estimation as reference**.** (H) Time‐dependent ROC curve analysis at 5 years of different predictors in the Zhejiang cohort.

To explore the association between *BAN* and GC metastasis, we examined *BAN* expression in GC tissues with metastasis compared to those in tissues without metastasis. Our qRT‐PCR analysis revealed that increased expression of *BAN* was significantly associated with GC metastasis and poor prognosis in the Zhejiang cohort (Fig. 7A,B). Multivariate Cox analysis further revealed that *BAN* expression was an independent predictor for assessing the prognosis of GC patients (Fig. 7C). Importantly, we also found that *BAN* expression levels were positively correlated with that of *BCAM* in GC tissues from the Zhejiang cohort (Fig. 7D,E). Kaplan–Meier curve analysis showed that high expression of both *BCAM* and *BAN* was correlated with the worse prognosis of GC patients, and GC patients with low expression levels of both *BCAM* and *BAN* had relatively longer survival time (Fig. 7F). To evaluate the potential clinical value of *BCAM* and *BAN* for prognosis, we computed their accuracy by PE curves compared with the American Joint Committee on Cancer (AJCC) staging system. Our data displayed that the prediction of GC prognosis using the combination of *BAN*, *BCAM,* and AJCC stage had the lowest predicting error in the Zhejiang cohort (Fig. 7G). Furthermore, the time‐dependent receiver operator characteristic (ROC) curve at 5 years showed that the area under the curve of *BAN* and *BCAM* combining with AJCC stage was higher than that of any other factors (Fig. 7H). Taken together, these data indicate that the combination of *BAN*, *BCAM,* and AJCC stage is more precise in predicting clinical outcome of GC patients.

## 4 Discussion

Since the mechanism of GC metastasis is still not fully understood, there is always a lack of effective GC metastasis treatment strategies and prognostic markers (Song *et al.*, 2017). Here, we systematically screened the key genes involved in GC metastasis and the prognosis of GC patients. We found that *BCAM* and its sense lncRNA *BAN* were significantly increased in GC tissues with metastasis and correlated with the reduced survival time of GC patients. BCAM promoted GC metastasis and was regulated by *BAN*. Moreover, our ROC analysis showed that both *BAN* and *BCAM* integrating with the AJCC staging might be a better prognostic predictor for GC patients than that of the only AJCC staging.

Recent studies revealed that miR‐199a‐5p and the 14‐3‐3beta‐FBI1/Akirin2 complex were involved in the regulation of BCAM expression. In a previous report, miR‐199a‐5p was found to repress BCAM expression by directly targeting its 3′UTR in human keratinocytes (Kim *et al.*, 2015). BCAM was also suggested as a target gene of the oncogenic 14‐3‐3beta‐FBI1/Akirin2 complex (Akiyama *et al.*, 2013). The 14‐3‐3beta‐FBI1/Akirin2 complex bound to the BCAM promoter and repressed BCAM transcription. In our study, we found that an uncharacterized lncRNA *BAN,* which is located in the genomic locus of *BCAM*, modulated BCAM expression. The knockdown of *BAN* suppressed BCAM expression at both the mRNA and protein levels. Our previous study reported that ephrin A1 expression was modulated by its sense lncRNA *GMAN*, which promoted the translational expression of ephrin A1 by competitively binding its antisense RNA *GMAN‐AS* (Zhuo *et al.*, 2019). It is unknown whether there is also an antisense RNA or miRNA related to *BAN* and BCAM. Moreover, knockdown of *BAN* reduced the stability of BCAM mRNA transcripts and BCAM protein. Considering the localization of BAN both in cytoplasm and nucleus, it is possible that there may exist different mechanisms for *BAN* to modulate BCAM expression. However, the detail molecular mechanisms were needed to be further investigated.

The previous study has shown that BCAM plays a functional role in the metastatic spreading of KRAS‐mutant colorectal cancer. Inhibition of BCAM impaired adhesion of KRAS‐mutant colorectal cancer cells specifically to endothelial cells (Bartolini *et al.*, 2016). The exact mechanism of BCAM regulating GC invasion and metastasis should be further explored. However, the KO of *BCAM* significantly reduced GC cell metastasis in a mouse model. These data suggest that BCAM may act as a promising target for GC metastasis treatment.

In our study, *BCAM* and *BAN* were significantly upregulated in GC tissues with metastasis and associated with poor prognosis of GC patients. *BCAM* and *BAN* may be independent prognostic factors for GC patients according to multivariate Cox analysis. Furthermore, integrating the expression of *BCAM* and *BAN* with AJCC staging showed more sensitivity and specificity in predicting GC prognosis based on both PC and ROC analysis. Taken together, our data indicate that *BCAM* and *BAN* might be used as prognostic biomarkers for GC patients in clinical practice. Undoubtedly, the possibility of *BCAM* and *BAN* for the prognostic prediction and treatment of GC needs to be further explored.

## 5 Conclusions

This study reveals that the upregulation of *BCAM* and its sense RNA *BAN* are significantly associated with GC metastasis and a shorter survival time of GC patients. KO of *BCAM* reduced GC cell invasion and metastasis. Knockdown of *BAN* not only inhibits *BCAM* expression, but also inhibits the migration and invasion of GC cells, which is effectively rescued by the ectopic expression of BCAM. Our data indicate that BCAM and *BAN* might be prognostic biomarkers for GC patients.

## Conflict of interest

The authors declare no conflict of interest.

## Author contributions

JJ, WZ, and TZ designed the experiments and interpreted the results, and performed research, and wrote the paper. JJ, ZH, KC, DG, XR, QS, YD, YL, SX, JD SL, and JW performed experiments. JJ, SX, ZH, DG, XR, YL WC, and WZ contributed valuable discussions on the project and participated in the manuscript preparation. TZ and WZ supervised the entire project. All authors approved the final version of the manuscript.

## Supporting information


**Fig. S1.** The expression of BCAM and BAN in gastric cancer cell lines.

**Fig. S2.** The localization of BAN in gastric cancer cells.

**Fig. S3.** The effects of BAN knockdown on the half‐life (t1/2) of BCAM protein.

**Data S1** **. **Supplementary Methods.
