# Supplementary figures and images for: Upregulation of BCAM and its sense lncRNA BAN are associated with gastric cancer metastasis and poor prognosis
Source: Mol Oncol. 2020 Feb 13;14(4):829–45. doi: 10.1002/1878-0261.12638 (PMC7138403; doi:10.1002/1878-0261.12638)

A

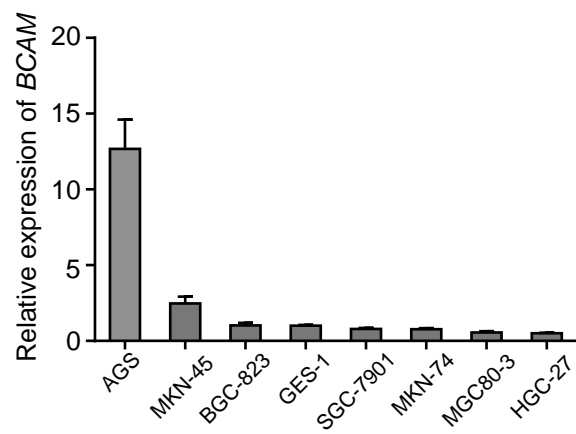

B

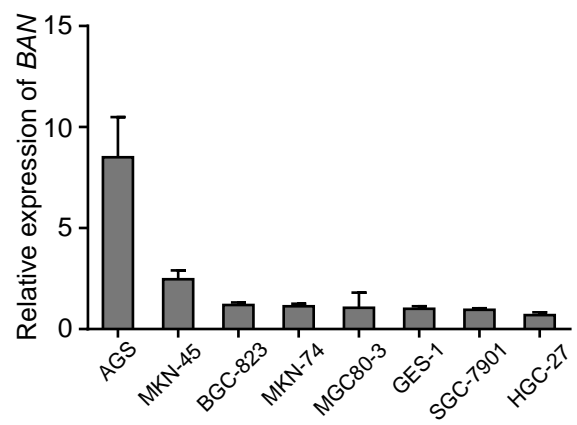

Supplement: Supplementary file 1 — Fig. S1. The expression of BCAM and BAN in gastric cancer cell lines. [file MOL2-14-829-s001.pdf]

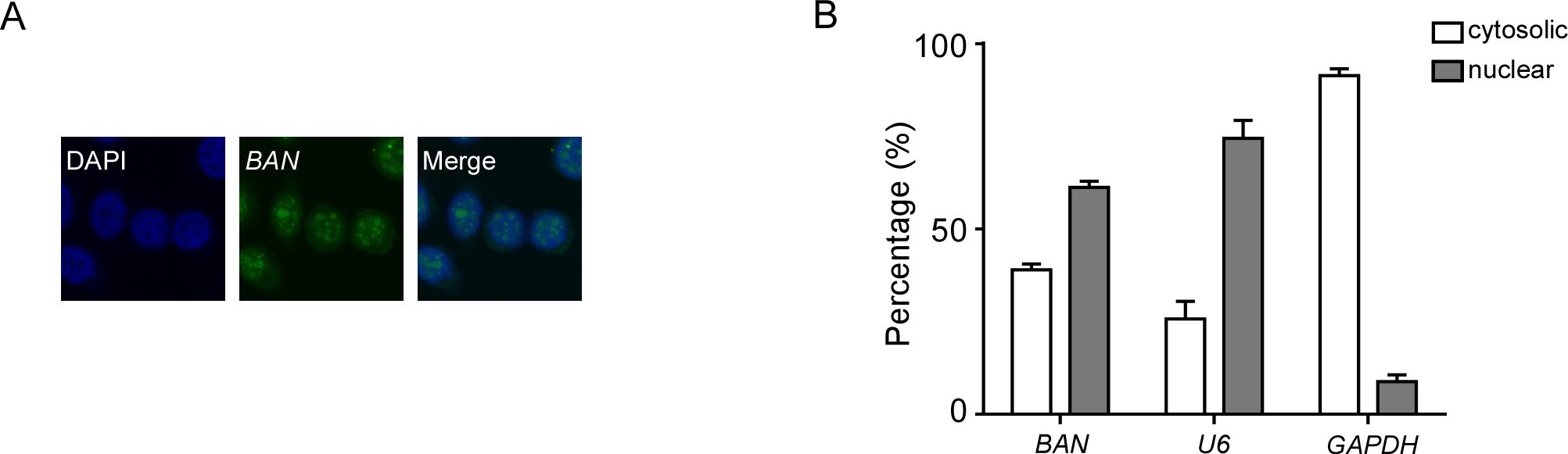

Supplement: Supplementary file 2 — Fig. S2. The localization of BAN in gastric cancer cells. [file MOL2-14-829-s002.tif]

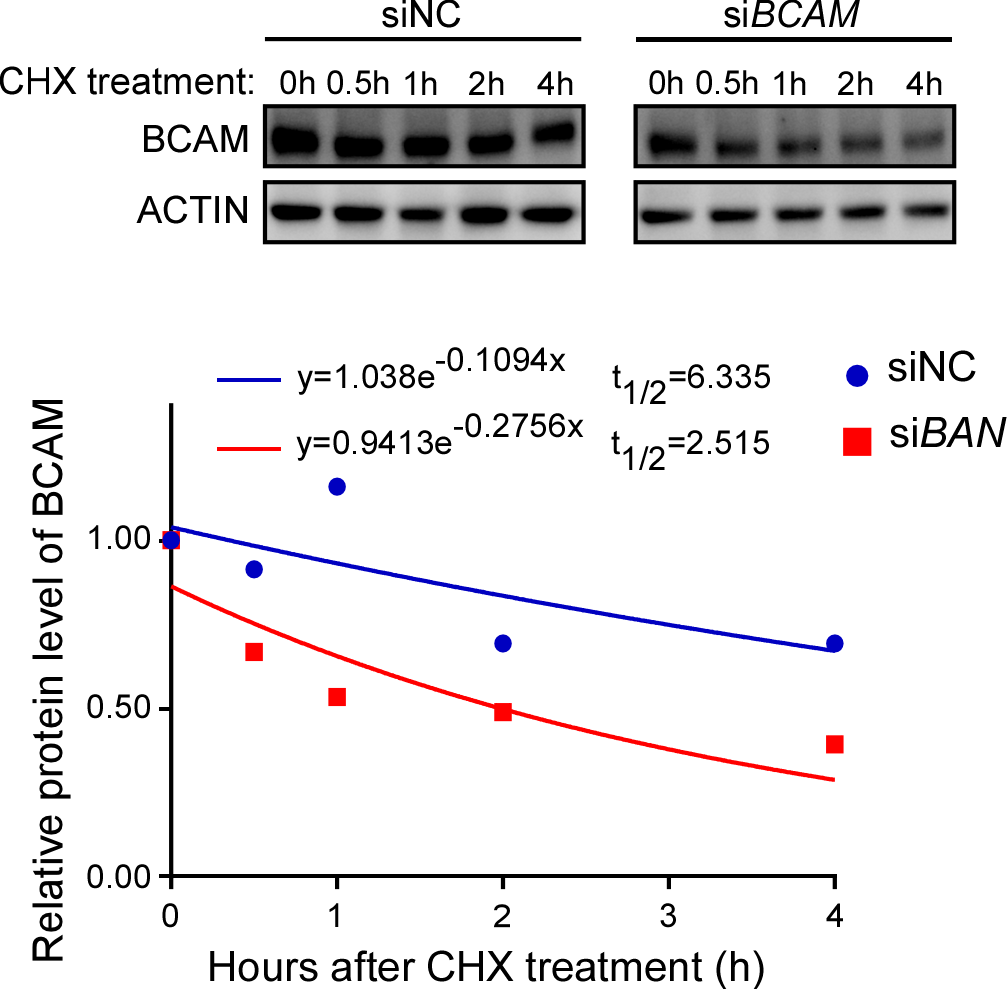

Supplement: Supplementary file 3 — Fig. S3. The effects of BAN knockdown on the half‐life (t1/2) of BCAM protein. [file MOL2-14-829-s003.tif]
