# Supplementary material for: Upregulation of BCAM and its sense lncRNA BAN are associated with gastric cancer metastasis and poor prognosis
Source: Mol Oncol. 2020 Feb 13;14(4):829–45. doi: 10.1002/1878-0261.12638 (PMC7138403; doi:10.1002/1878-0261.12638)
Supplement: Supplementary file 4 — Data S1 . Supplementary Methods. [file MOL2-14-829-s004.doc]

**Supplementary Methods**

***In vitro* transcription**

Full length human *BAN* was cloned into the pCS107 vector, which contains the SP6 and T7 promoters. Fluorescein-labeled RNA was transcribed from the linearized pCS107-*BAN* plasmid using fluorescein RNA labeling mix (Roche, Switzerland), RNA inhibitor (Invitrogen, USA), and SP6 or T7 RNA polymerase (Roche, Switzerland) according to the manufacturer’s instructions. The transcripts were purified with RNeasy Mini Spin Column (Qiagen, Germany).

**RNA fluorescence in situ hybridization**

Cells were washed in PBS and fixed with 4% paraformaldehyde. The fixed cells were permeabilized with 0.5% Triton X-100, followed by washing 3 times with PBS. The cells were preincubated with hybridization buffer and were hybridized with fluorescein-labeled RNA probe in hybridization solution at 37°C. The cells were washed 3 times with 2 x SSC/50% methylamide at 50°C, and then 3 times with 2 x SSC at 50°C. The cells were incubated with DAPI and mounted with Gold Antifade Reagent (Invitrogen, USA). The images were obtained with an IX81-Fv1000 confocal microscope (Olympus, Japan).

**RNA stability assay**

The mRNA stability in vivo is normally reported as the time for degrading half of the existing mRNA molecules. Cells were cultured in 12-well plates. With 48 hours post transfection, actinomycin D was added at 6 hours, 4 hours, 1 hours and 0 hour before cell collection. Total RNA was extracted and analyzed by qRT-PCR.

**RNA degradation assay**

Cells were cultured in 6-well plates. With 48 hours post transfection, CHX was added at 4 hours, 2 hours, 1 hours, 0.5 hours and 0 hour before cell collection. Cells were lysed using RIPA protein extraction reagent (Beyotime, China) supplemented with a protease inhibitor cocktail (Roche, USA). The cell lysates were separated by SDS-PAGE and were transferred onto a PVDF membrane. The membranes were blocked with bovine serum albumin (BSA, 5%) for 1 h at room temperature. The proteins were detected using an anti-BCAM monoclonal antibody (1:1000, Abcam, UK) and anti-ACTIN (1:1000, Sigma-Aldrich, USA).

**Supplementary Figure Legends**

**Fig. S1.** The expression of BCAM and *BAN* in gastric cancer cell lines. (A, B) qRT-PCR analysis of *BCAM* (A) and *BAN* (B) expression in gastric cancer cell lines. Data are presented as the means ± SDs.

**Fig. S2.** The localization of *BAN* in gastric cancer cells. (A) RNA-FISH analysis of the localization of *BAN* in BGC-823 cells. (B) The expression of *BAN* in the nuclear and cytosolic fractions. Data are presented as the means ± SDs.

**Fig. S3.** The effects of *BAN* knockdown on the half-life (t_1/2_) of BCAM protein. Knockdown of *BAN* decreased the half-life of BCAM protein.
